# IDENTIFICATION OF PATIENT PORTAL MESSAGES SENT FROM NON–PROXY ACCOUNT CARE PARTNERS AND THEIR PORTAL USE PATTERNS

**DOI:** 10.1093/geroni/igae098.0659

**Published:** 2024-12-31

**Authors:** Jennifer Portz, Daniel Martin, Kelly Gleason, Karla Washington, Andrea Daddato, Dave Powers, Jason Lyons, Elizabeth Bayliss

**Affiliations:** University of Colorado, Aurora, Colorado, United States; Kaiser Permanente Colorado, Aurora, Colorado, United States; Johns Hopkins University, Baltimore, Maryland, United States; Washington University in St. Louis, St. Louis, Missouri, United States; Kaiser Permanente Colorado, Aurora, Colorado, United States; Kaiser Permanente Colorado, Aurora, Colorado, United States; Kaiser Permanente Colorado, Aurora, Colorado, United States; Kaiser Permanente Colorado, Aurora, Colorado, United States

## Abstract

Automating care partner identification via the electronic health record (EHR) can streamline the delivery of evidence-based caregiving interventions through patient portals and is vital for supporting care partners of people living with dementia (PLWD). This retrospective cohort study focused on identifying patient portal messages from non-proxy care partners and exploring associated portal usage. Conducted at Kaiser Permanente Colorado, it analyzed data from 789 PLWD aged ≥60, utilizing the EHR and Virtual Data Warehouse. Structured patient contact data (e.g., emergency contact, family contact) and unstructured portal notes were used to match contact names with caregiver terms, employing a rule-based algorithm to identify care partner messages. Results showed that 95% of PLWD had at least one care partner listed, with an average of 2.1. A majority (77%) engaged with the patient portal, and 57% had messages sent by care partners, predominantly non-proxy (7% were from proxy accounts). Non-proxy care partners sent an average of six messages during the study period. Additionally, 54% of PLWD had portal activity on days when care partners sent messages, showing their involvement in healthcare management. Care partners mainly accessed clinical visit information, medication details, lab scheduling/results, and primary care provider information on these days. This study underscores the importance of automated care partner identification and non-proxy engagement via patient portals, facilitating better support for PLWD and enhancing their care partners’ involvement.

